# Skeletal muscle oxidative stress and inflammation in aging: Focus on antioxidant and anti-inflammatory therapy

**DOI:** 10.3389/fcell.2022.964130

**Published:** 2022-08-30

**Authors:** Mingming Chen, Yiyi Wang, Shoulong Deng, Zhengxing Lian, Kun Yu

**Affiliations:** ^1^ College of Animal Science and Technology, China Agricultural University, Beijing, China; ^2^ Zhejiang A&F University, Zhejiang Provincial Key Laboratory of Characteristic Traditional Chinese Medicine Resources Protection and Innovative Utilization, Lin’an, China; ^3^ NHC Key Laboratory of Human Disease Comparative Medicine, Institute of Laboratory Animal Sciences, Chinese Academy of Medical Sciences and Comparative Medicine Center, Peking Union Medical College, Beijing, China

**Keywords:** skeletal muscle, aging, oxidative stress, inflammation, treatment strategy

## Abstract

With aging, the progressive loss of skeletal muscle will have negative effect on multiple physiological parameters, such as exercise, respiration, thermoregulation, and metabolic homeostasis. Accumulating evidence reveals that oxidative stress and inflammation are the main pathological characteristics of skeletal muscle during aging. Here, we focus on aging-related sarcopenia, summarize the relationship between aging and sarcopenia, and elaborate on aging-mediated oxidative stress and oxidative damage in skeletal muscle and its critical role in the occurrence and development of sarcopenia. In addition, we discuss the production of excessive reactive oxygen species in aging skeletal muscle, which reduces the ability of skeletal muscle satellite cells to participate in muscle regeneration, and analyze the potential molecular mechanism of ROS-mediated mitochondrial dysfunction in aging skeletal muscle. Furthermore, we have also paid extensive attention to the possibility and potential regulatory pathways of skeletal muscle aging and oxidative stress mediate inflammation. Finally, in response to the abnormal activity of oxidative stress and inflammation during aging, we summarize several potential antioxidant and anti-inflammatory strategies for the treatment of sarcopenia, which may provide beneficial help for improving sarcopenia during aging.

## Introduction

Skeletal muscle, as a powerful mediator of health and longevity, is the most massive plastic organ in the body, and plays a vital role in respiration, movement, metabolism, daily physical activity, protection, and maintenance of posture and body balance (Frontera and Ochala, 2015). The progressive loss of skeletal muscle with aging will have negative effect on multiple physiological parameters, such as exercise, respiration, thermoregulation, and metabolic homeostasis. This age-related muscle wasting, known as sarcopenia, is a long-term process caused by a number of complex factors in which the average muscle capacity decreased during aging, resulting in increased opportunities of instability, falls, and fractures (Tieland et al., 2018). In aging skeletal muscle, reduced biosynthesis, slower metabolism, and smaller mitochondrial size cause rapid loss of skeletal muscle mass and efficiency parameters. It is typically characterized by weakened and reduced muscle mass and fiber cross-sectional area, the changes in myosin isotype or fiber type, and the net loss of cellular components such as organelles, cytoplasm, and total proteins (Chemello et al., 2011; Yin et al., 2021). It is manifested as decreased muscle strength, easy fatigue, and decreased exercise capacity.

Although it has a profound impact, the molecular mechanisms causing skeletal muscle changes with age are not completely clear. Current evidence suggests that sarcopenia is regulated by a complex network. Mechanistically, sarcopenia involves several pathophysiological processes, including oxidative stress, inflammation, mitochondrial dysfunction, and subsequent activation or inhibition of signal transduction, such as the mTOR signal pathway, autophagy lysosome system, and ubiquitin-proteasome system. Specifically, the abnormal production of reactive oxygen species (ROS), chronic and low-grade inflammation, mitochondrial dysfunction, modified protein synthesis, synthetic metabolic passivation, denervation and reduced muscle regeneration are some of the key processes associated with sarcopenia (Cuthbertson et al., 2005; Breen and Phillips, 2013; Vasilaki and Jackson, 2013; Almada and Wagers, 2016; Pollock et al., 2017; Sakellariou et al., 2017; Scalabrin et al., 2019). During these processes, the excessive production of ROS, which controls redox signal pathway in muscle fibers, can cause oxidative damage and mitochondrial dysfunction, attenuate ATP production, increase protein degradation and reduce protein synthesis, further resulting in muscle loss and strength decline (Powers, 2014). Furthermore, the elevated inflammation causes oxidative stress and anabolic resistance, leading to muscle loss. For example, the chronic and low-grade inflammation affects the metabolism of skeletal muscle cells through interactions among various cytokines. IL-6 secreted by skeletal muscle activates the STAT3 protein through inhibiting JAK/STAT3, PI3K/Akt, and ERK signaling pathways, and plays a crucial role in protein degradation of skeletal muscle (Silva et al., 2015). In addition, accumulation of fat and fibrosis can lead to poor muscle mass, and the reduced capacity for self-renewal and differentiation of skeletal muscle satellite cells cause impaired muscle regeneration.

In short, oxidative stress and inflammation are important factors that are closely associated with aging. Here, we detailed elaborate on the relationship between aging and sarcopenia, as well as aging and oxidative stress, and clarify that aging-mediated skeletal muscle oxidative stress and oxidative damage are direct factors in the development of sarcopenia. Specifically, ROS-mediated skeletal muscle mitochondrial dysfunction is a potential gatekeeper for skeletal muscle aging. Excessive ROS production in aging skeletal muscle causes mitochondrial dysfunction and further aggravates inflammation. Finally, we summarize several potential antioxidant and anti-inflammatory strategies that are expected to be used to treat sarcopenia, although to date, there is no effective or approved drug therapy.

## Aging-mediated oxidative stress and oxidative damage in skeletal muscle

### Aging and sarcopenia

Aging is a natural process related to the physical function deterioration. The progressive loss of muscle mass, strength and function is one of the most prominent signs of aging. This skeletal muscle degeneration associated with aging can lead to adverse consequences and seriously damage the patient’s quality of life. The geriatricians defined sarcopenia as “the loss of skeletal muscle function and mass associated with age”, and launched the newest diagnostic standards for sarcopenia, which including low muscle strength, diminished muscle quantity, reduced muscle quality or weak physical performance (Cruz-Jentoft et al., 2019). Sarcopenia is currently recognized as a serious geriatric problem and an important condition for predicting frailty in the elderly (Cruz-Jentoft et al., 2019). Muscle mass gradually decline from 30 to 40 years old as muscle funcition weakens (Denison et al., 2015). This loss of muscle mass is more severe in people with a sedentary or inactive lifestyle. From 50 to 60 years of age, the loss of muscle mass may reache 1%–2%year and 3–5%year for the elderly (Denison et al., 2015). Of these individuals, 30%–50% of their muscle mass may be completely disappear from the ages of 40–80 (Denison et al., 2015). As a multi-faceted elderly disease characterized by both progressive and systemic loss of muscle mass and function (Cruz-Jentoft and Sayer, 2019; Borsch et al., 2021), and the development and progression of sarcopenia is consistently accompanied by a variety negative consequences, including falls, broken bones, loss of movement and even death (Morley et al., 2011; Bischoff-Ferrari et al., 2015; De Buyser et al., 2016). Similar to any other complex syndrome, the pathogenesis and progression of sarcopenia are diverse. Although the potential mechanism of sarcopenia remains elusive, a number of age-related factors can lead to the deterioration of the structure and function of skeletal muscle, thereby resulting in sarcopenia. For example, skeletal muscle satellite cell dysfunction, imbalance in protein turnover, adipose tissue infiltration, oxidative stress, mitochondrial dysfunction, inflammation, insulin resistance, capillary, and neuromuscular injury (Meng and Yu, 2010; Fulop et al., 2017; Pagano et al., 2018). The detailed biological mechanism of aging-induced sarcopenia can be referred to other excellent review (Englund et al., 2021; Kim et al., 2021), which will not be repeated here.

### Aging and oxidative stress

Of all the potential etiological bases of sarcopenia, ROS production, correlated oxidative damage, and shortcomings in redox signaling have been repeatedly proved to be closely related to various forms of muscle pathophysiology during aging (Jackson, 2016; Damiano et al., 2019). For example, muscle fibers isolated from older mice generate more ROS than young adult mice (Englund et al., 2021). During aging, ROS basal levels are increased in muscles and satellite cells (Minet and Gaster, 2012; Palomero et al., 2013), and high levels of ROS have been proved to enhance DNA damage, mitochondrial dysfunction and protein damage. Specifically, elevated ROS levels cause oxidation of DNA, proteins, and lipids; increased protein carbonylation; impaired myogenic proteins and autophagy process; and inhibited skeletal muscle cell differentiation (Scherz-Shouval et al., 2007; Sandiford et al., 2014; Sakellariou et al., 2016). However, with aging, more ROS is generate even the cells in quiescent state, which mainly from mitochondria and NADPH oxidase (NOX) (Jackson, 2016; Damiano et al., 2019). Accumulated ROS also trigger apoptotic signaling cascades with aging (Meng and Yu, 2010; Barbieri and Sestili, 2012). It is reported that activation of mitochondrial cystathione non-dependent apoptosis, cystathione 2 and JNK-mediated apoptosis leading to age-related muscle loss (Braga et al., 2008; Marzetti et al., 2008). Interestingly, ROS is the cause of neuromuscular junction dysfunction in myopenia. The more ROS are produced in the neuromuscular junction region of older mice, which is related to the decrease of neurotransmitter release (Ivannikov and Van Remmen, 2015). This may lead to impaired action potential generation and result in reduced muscle strength associated with sarcopenia. In addition, ROS may boost anabolic resistance in sarcopenia at multiple levels. Increased levels of ROS, such as H_2_O_2_, can suppress the phosphorylation of mTOR, Akt, and downstream targets p70S6K and 4E-BP1 (Powers et al., 2016; Gomez-Cabrera et al., 2020). The common compensatory response to ROS accumulation is the enhanced antioxidant defenses of cells (Ji, 2015). Most studies showed increased activity of superoxide dismutase 1 (SOD1) (Sullivan-Gunn and Lewandowski, 2013), superoxide dismutase 2 (SOD2) (Gianni et al., 2004), catalase (CAT) (Gianni et al., 2004; Palomero et al., 2013; Sullivan-Gunn and Lewandowski, 2013), and glutathione peroxidase (GPx) (Palomero et al., 2013), and it has observed age-dependent alterations in antioxidant enzyme activity in muscle (Ji, 1993). Although antioxidant enzyme activity increases with aging, this compensatory adaptation mechanism does not fully offset the increase in oxidative stress. The excessive ROS generated in aging muscles might restrain the key components of the Akt/mTOR pathway, thus limiting their response to exercise stimulation (Powers et al., 2016; Gomez-Cabrera et al., 2020). In fact, decreasing oxidative stress during aging could enhance exercise adaptability. For instance, antioxidant supplements can restore the stimulation of protein synthesis by age-related deficient leucine in rats (Marzani et al., 2008). The above mentioned evidence suggests that excessive ROS-induced oxidative stress can inhibit protein synthesis during sarcopenia.

As one of the key events of aging, muscle loss affects the overall cellular homeostasis. Muscle plasticity allows muscles to tolerate any stress-related biological alteration. However, aging also affects muscle malleability in terms of energy intake and consumption (Distefano and Goodpaster, 2018). ROS-mediated oxidative stress is a common phenomenon under the conditions of energy intake and consumption, which may be a significant target of age-related complications. Skeletal muscle satellite cells can maintain the ability of muscle adaptation and regeneration, which is important to delay or prevent aging. The quiescent satellite cells rely on glycolysis to produce energy, which is conducive to maintaining low levels ROS in satellite cell pool and keeping the redox system in an active state (Radak et al., 2008; Thirupathi et al., 2020). However, the damage of the satellite cell cycle by ROS-induced oxidative stress may lead to the failure of quiescent satellite cells to activate in the process of aging, thereby effecting the ability of muscle regeneration and muscle contraction. In addition, studies have demonstrated that aged muscle have impaired regenerative capability and muscle functions (Joanisse et al., 2016; Pisot et al., 2016). After muscle atrophy, aged muscle also fail to regenerate, while young muscle tissue can recover (Verdijk et al., 2009). Although the mechanism of this regeneration process is still elusive, ROS-induced oxidative stress may be one of the reasons for this failure in muscle growth capacity. However, it should be noted that it remains unclear whether ROS can act as a direct signal transduction in the case of aging. In addition, only few genetic manipulations aimed at reducing ROS activities have led to increased lifespan in mammals, suggesting that oxidative stress may play a limited role in aging but that reducing oxidative stress delays lesions (Schriner et al., 2005; Yoshida et al., 2005; Salmon et al., 2010).

## ROS may be a potential gatekeeper of mitochondrial dysfunction in aged skeletal muscle

Much of the interest in ROS and aging involves the potential role of mitochondria as a resource and target of ROS (Jang and Van Remmen, 2009; Salmon et al., 2010). Skeletal muscle fibers are enriched with mitochondria whose function is to synthesize ATP through the oxidative phosphorylation to supply energy for muscle contraction. Accumulating evidence have showed that mitochondrial dysfunctional may play a core role in the pathogenesis of sarcopenia, while maintaining the normal mitochondrial function is crucial for skeletal muscle development (Calvani et al., 2013; Bhatti et al., 2017).

The maintenance of mitochondrial function is a dynamic balance process of mitochondrial quality control, which is an elaborate and complex network in eukaryotic cells. Specifically, it maintain mitochondrial homeostasis through four key processes: mitochondrial protein homeostasis, biogenesis, kinetics and autophagy. The mitochondrial dysfunction magnified by defects in the quality control process is increasingly considered to be the main pathophysiological mechanism of sarcopenia. Skeletal muscle mitochondria are interconnected to form a dynamic network, and their composition and morphology can be changed by the dynamic regulation of mitochondrial fission and fusion (Ogata and Yamasaki, 1997; Picard et al., 2011; Hood et al., 2019). Among them, mitochondrial fusion is regulated by MFN1/2 and OPA1, while mitochondrial fission is regulated by DRP1 and FIS1. A recent study has demonstrated that the morphology and dynamics of mitochondria changed during aging (Leduc-Gaudet et al., 2015). Evidence has demonstrated that the level of FIS1 and the activity of DRP1 decrease in aging cells, which may affect satellite cell activation and skeletal muscle regeneration (Mai et al., 2010). In mice, muscle aging is associated with increased myofibrillar mitochondrial complexity and expansion of mitochondria under the sarcolemma (Leduc-Gaudet et al., 2015). The MFN2/DRP1 ratio increased significantly in aged mice, suggesting an increase in mitochondrial fusion capacity during skeletal muscle aging (Leduc-Gaudet et al., 2015). In short, the control of mitochondrial dynamics may represent a mechanism of skeletal muscle aging, leading to the accumulation of mitochondrial dysfunction during aging, thereby leading to the development of sarcopenia. Furthermore, recent studies suggest that the alterations in mitophagy may be associated with the development of sarcopenia. It has been found that the Parkin-PINK1 mitophagy regulatory axis may be altered during muscle aging in humans, suggesting a phenomenon of mitophagy deficiency during muscle aging (Gouspillou et al., 2013; Drummond et al., 2014; Gouspillou et al., 2014). In addition, the voltage-dependent anion channels (VDAC) help to recruit Parkin to dysfunctional mitochondria, which is a necessary step to start mitochondrial autophagy (Faitg et al., 2017). However, the Parkin/VDAC ratio appears to be reduced in muscle of older men (Gouspillou et al., 2014). A recent study demonstrated that overexpression of Parkin in *Drosophila* can increase its lifespan, which is related to the increase of citrate synthase activity and the decrease of protein aggregates in aging muscle (Rana et al., 2013). Taken together, these results suggest that the mitophagy mechanisms are altered and autophagic potential is reduced during muscle aging. And such alterations would represent an attractive cellular mechanism to explain the accumulation of dysfunctional mitochondria during muscle aging.

In fact, among the various theories that have been proposed to explain sarcopenia, the mitochondrial aging theory assumes that the accumulation of mitochondrial dysfunction plays a causal role in muscle atrophy with aging. This theory is generally accepted that oxidative damage to mitochondrial proteins, lipids and DNA will accumulate with aging due to the generation of ROS inherent in the activity of the respiratory chain (Faitg et al., 2017). The ROS produced by mitochondria are also critical for maintaining muscle functions such as skeletal muscle development, muscle mass, mitochondrial biogenesis and injury repair. However, the excessive ROS will cause oxidative damage to many cell molecules and cell structures, and the accumulation of this damage is the root of cellular dysfunction and eventually leads to cellular aging (Harman, 1956). Moreover, this oxidative damage is considered to exacerbate the production of mitochondrial ROS, weaken the ability of mitochondria to adequately match cellular ATP demands and trigger mitochondria-mediated apoptosis (Faitg et al., 2017). Multiple studies agree that muscle aging is related to an increase of the oxidative stress markers (Chabi et al., 2008; Sinha-Hikim et al., 2013). In addition, several studies have also reported increased ROS production by muscle mitochondria during aging (Capel et al., 2005; Dirks et al., 2006; Chabi et al., 2008; Lanza et al., 2012). A study indicated that over-expressed CAT in mitochondria attenuated the adverse effects of aging on muscle strength in mice (Umanskaya et al., 2014). A recent study revealed that the mitochondrial calcium uptake family member 3 (MICU3) expression was down-regulated during aging in skeletal muscle, which is correlated with reduced myogenesis but elevated oxidative stress and apoptosis (Yang et al., 2021). Conversely, MICU3 reconstitution enhances antioxidants, prevents the accumulation of mitochondrial ROS, reduces apoptosis and increases myogenesis (Yang et al., 2021). However, it should be noted that although most studies agree that muscle aging is associated with the occurrence of oxidative stress, the involvement of mitochondria in this process is still unclear.

The differentiation of skeletal muscle satellite cells (SMSCs) into myotubes and further fusion into myofibers are decisive steps in muscle regeneration. Evidence suggests that increased ROS negatively affects the regenerative capacity of SMSCs during aging, which is partly mediated by increased ROS-induced oxidative stress. Specifically, the proliferation and differentiation of SMSCs decrease with aging (Dalle et al., 2017), and more SMSCs enter a permanent aging stage from reversible quiescent state in aging muscle (Sousa-Victor et al., 2014). At the same time, the antioxidant capacity of SMSCs also decreases with aging (Fulle et al., 2005), and what is worse, the production of ROS continuously increases during this process (Minet and Gaster, 2012). However, the exact mechanism of ROS-mediated reduction of SMSCs regeneration capacity during aging remains unclear. Current evidence indicates that multiple redox sensitive signaling in SMSCs are altered during aging, such as p38/MAPK, Wnt, Notch, and JAK/STAT3 (Szentesi et al., 2019; Foreman et al., 2021). It is unclear why these pathways are altered with aging, and why they no longer adapt to aging. Although aged SMSCs produce more ROS, it is conceivable that the inhibition of ROS could restore the proliferation and regeneration capabilities of aged SMSCs (Garcia-Prat et al., 2016).

## Skeletal muscle aging and oxidative stress mediated inflammatory response

A systemic, chronic, low-grade, sterile inflammatory state in aging was termed inflammatory aging (Cevenini et al., 2013). The inflammatory aging contributes to changes in skeletal muscle properties and sarcopenia, and mediates Alzheimer’s disease and cognitive deficits in the elderly (Dalle et al., 2017; Zembron-Lacny et al., 2019). Inflammation is highly prevalent phenomenon in the elderly populations and is typified by increased levels of blood inflammatory markers in tissues and cells, leading to an increasing risk of chronic disease, weakness, disability and early death (Ferrucci and Fabbri, 2018). Inflammatory cells and muscle cells can generate inflammatory cytokines, contribute to the occurrence and development of sarcopenia, which is characterized by lower muscle mass, weakened muscle strength, and worse physical function, leading to an increased possibility of adverse events. Inflammatory cytokines, including pro-inflammatory and anti-inflammatory cytokines interleukin 6 (IL-6) and tumor necrosis factor alpha (TNF-α), have been repeatedly proved to be closely associated with sarcopenia in humans and animals (Haddad et al., 2005; Dalle et al., 2017). Normally, the balance between skeletal muscle anabolism and catabolism is maintained by pro-inflammatory cytokines, but the expression of pro-inflammatory cytokines were increased during muscle atrophy, and leading to increased catabolism. Also, pro-inflammatory cytokines can inhibit skeletal muscle cells protein synthesis, impair muscle integrity and function, and thus lead to sarcopenia (Sharma and Dabur, 2020). The anti-inflammatory cytokines (such as IL-4, IL-10 and IL-15) can counteract the expression and activity of pro-inflammatory cytokines, especially IL-6 and TNF-α, to reduce muscle atrophy and delay sarcopenia (Marzetti et al., 2009; Hofmann et al., 2012). In addition, the anti-inflammatory cytokine, such as IL-4, can better glucose metabolism in muscle cells, and serve as a myogenic cells recruitment factor during muscle growth to promote muscle regeneration (Horsley et al., 2003; Heredia et al., 2013; Chang et al., 2019). The activation of the NF-κB pathway is a major trigger of inflammation in inactive muscle. The transgenic mice that overexpress IKKβ, the main activator of NF-κB, have up-regulated expression of MuRF1, leading to the appearance of muscle atrophy (Cai et al., 2004). On the contrary, the absence of p105/p50 subunits of NF-κB has been proved to have an inhibitory effect on muscle atrophy (Hunter and Kandarian, 2004). As mentioned above, NF-κB activation is mainly related to the excessive production of pro-inflammatory cytokines. Also, NF-κB can stimulate the expression of MuRF1, thereby degrading muscle contraction protein (Cai et al., 2004). In addition, TNF-α is the strongest activator of the NF-κB pathway, which contributes to a positive feedback loop via activation of NF-κB, thereby inducing TNF-α and driving NF-κB-mediated muscle atrophy (Cai et al., 2004).

As mentioned above, increased ROS is not a coincidence with age and sarcopenia. Oxidative stress, as an effective activator of macrophages and neutrophils, increases due to intense exercise, aging and ischemia (Bartnik et al., 2000). The phagocytic activity of macrophages and neutrophils leads to the generation and release of cytotoxic proteases, pro-inflammatory cytokines and free radicals, including reactive oxygen and nitrogen species (RONS) (Lu and Wahl, 2005). It is reported that the sensitivity of elderly rats to inflammatory injury increases, resulting in more pro-inflammatory cytokines, RONS and transcription factors (Chung et al., 2002). Among them, transcription factor NF-κB stimulates the expression of proinflammatory gene and regulates subsequent inflammation and muscle recovery from injury (Hnia et al., 2008). In aged rat muscle, age-related oxidative stress appears to be associated with NF-βB, macrophage infiltration and neutrophil activity (Ghaly and Marsh, 2010). In younger rodents, an appropriate inflammatory response is essential for muscle regeneration (Shen et al., 2008). It has been proved that this inflammatory response is regulated by the cyclooxygenase two and TGF-β pathways (Bondesen et al., 2004; Shen et al., 2008). Furthermore, the balance of notch-activation and TGF-β inhibition of satellite cells in aged muscle determines the differentiation of satellite cells into myogenic cells, which eventually dictates whether myogenesis and muscle regeneration in injured muscle can occur successfully (Carlson et al., 2008). Therefore, increased inflammatory response may lead to excessive TGF-β1 production, fibrosis scar formation and diminished muscle regeneration (Li et al., 2004).

In short, anti-inflammatory cytokines are a potential therapeutic target for sarcopenia. To maximize muscle regeneration efficiency, it is critical to minimize secondary inflammation of destructive leukocytes and maximize regeneration-promoting inflammatory cytokines and growth factors. However, studies on the interaction between sarcopenia and anti-inflammatory cytokines are very few, and need to be further investigated in depth.

**FIGURE 1:**
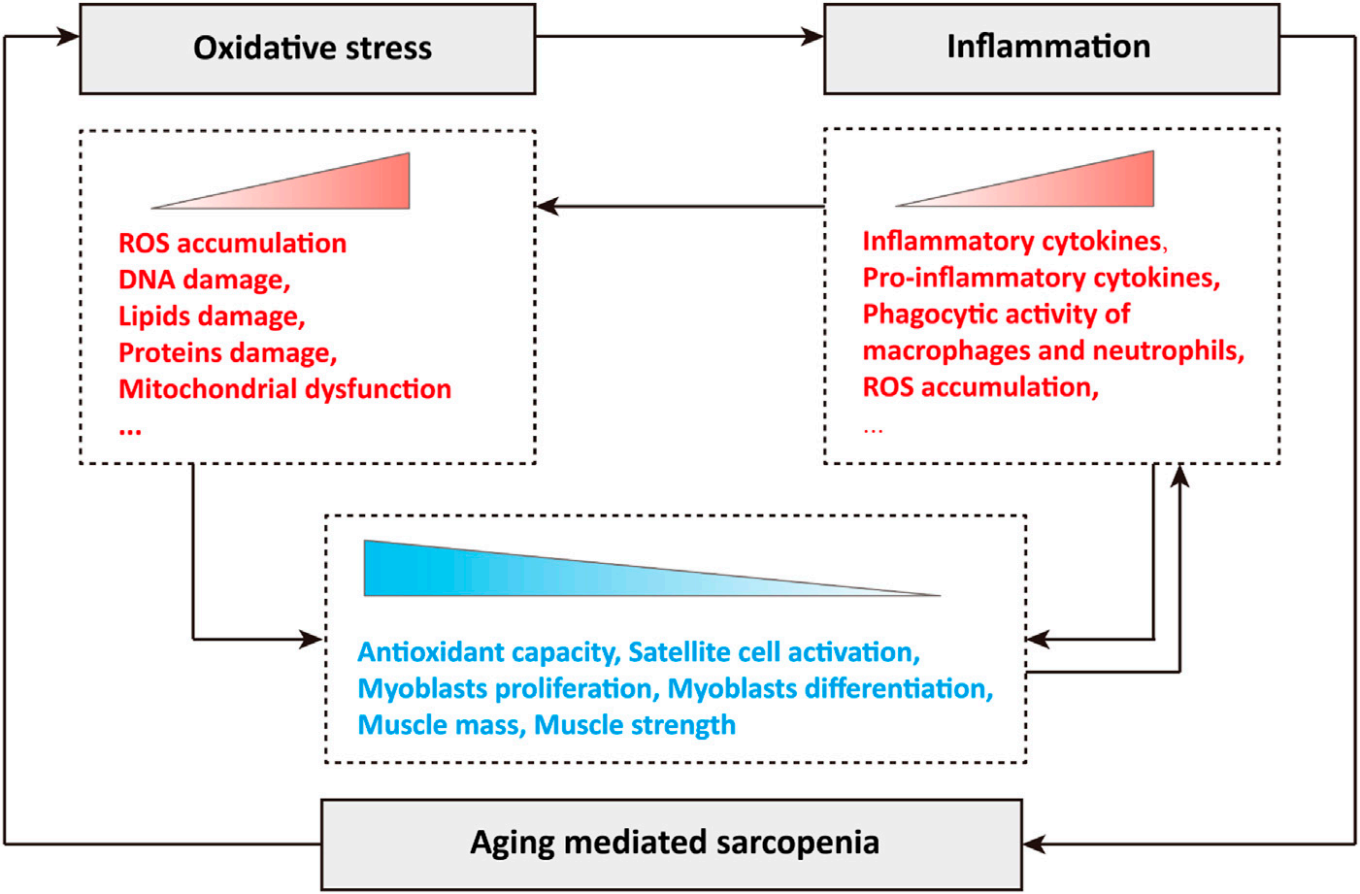
Relationship among oxidative stress, inflammation and sarcopenia during aging.

In conclusion, oxidative stress and inflammation play a crucial role in the occurrence and development of sarcopenia during aging. Briefly, the production of ROS leads to oxidative stress in cells, and subsequent oxidative damage, such as DNA damage, lipid damage and protein damage, disrupts mitochondrial dynamics and quality control, resulting in mitochondrial dysfunction (Figure 1). Oxidative stress stimulates the abnormal accumulation of inflammatory cytokines, especially pro-inflammatory cytokines, activates the phagocytic activity of macrophages and neutrophils, and leads to the production and release of cytotoxic protease, pro-inflammatory cytokines and ROS, and the excessive ROS further stimulates the occurrence of oxidative stress (Figure 1). During aging, the synergistic effects of oxidative stress and inflammation greatly reduce cellular antioxidant capacity, significantly inhibit the activation of skeletal muscle satellite cells, the proliferation and differentiation of myoblasts, and causing loss of muscle mass and muscle strength, which manifests as sarcopenia (Figure 1). In turn, sarcopenia during aging further exacerbates oxidative stress and inflammation, forming a poor vicious cycle (Figure 1).

## Potential therapeutic strategies for sarcopenia during aging

**FIGURE 2:**
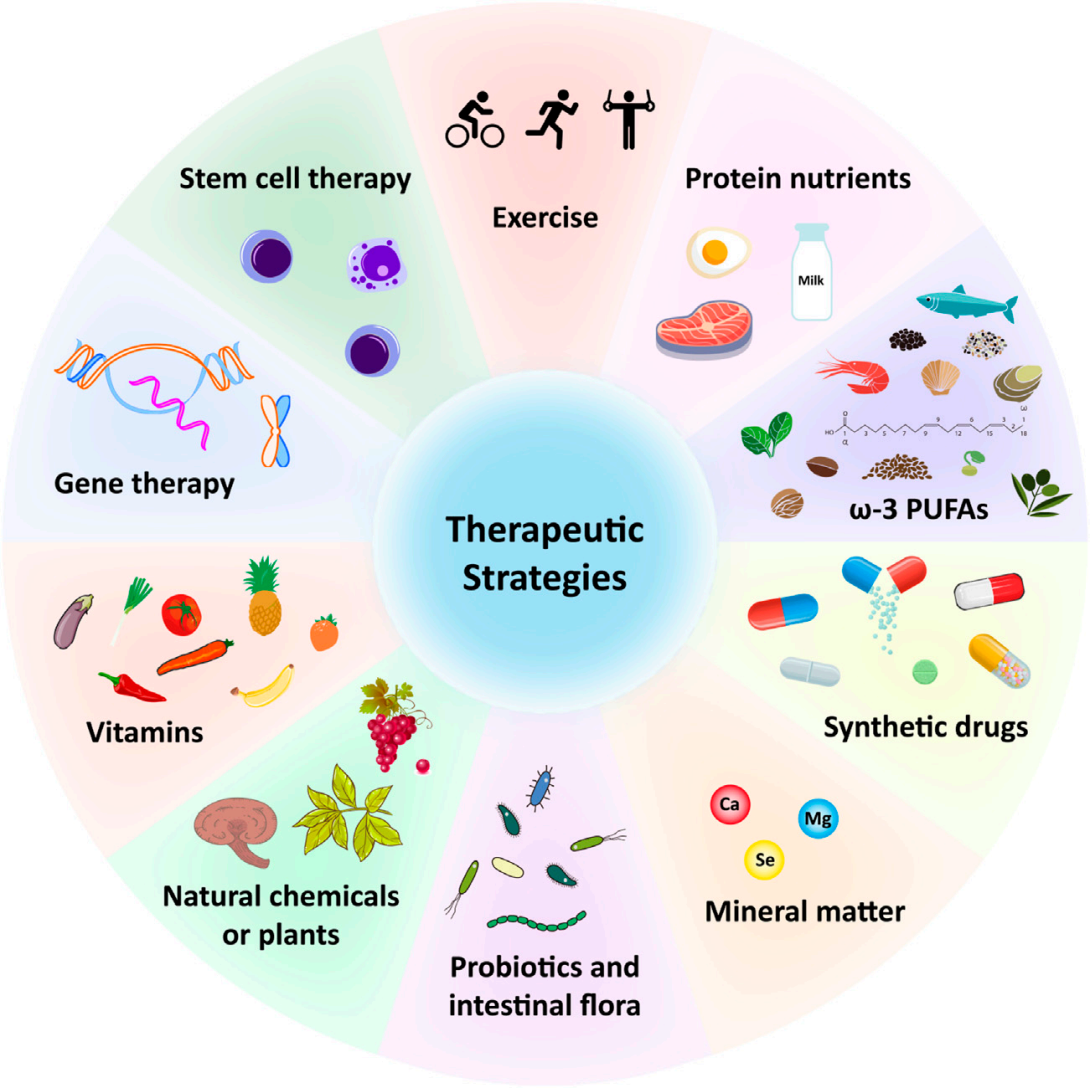
Potential therapeutic strategies for sarcopenia during aging. ω-3, Omega-3; PUFAs, Polyunsaturated fatty acids; Ca, Calcium; Mg, magnesium; Se, selenium.

Multiple therapeutic strategies have been attempted and have proven effective in treating sarcopenia during aging. Since it is affected by many factors, the combination of exercise, potential antioxidant therapy and anti-inflammatory effect represents probably the most efficient strategy to prevent or treat skeletal muscle aging and sarcopenia. A large number of antioxidant and anti-aging therapies have been proposed (Figure 2; Table 1). For example, protein, amino acid and peptide supplements are thought to be highly effective in enhancing muscle metabolism and protecting muscle wasting during a prolonged immobilization (Dardevet et al., 2012) (Table 1). The supplementation of minerals such as calcium, magnesium, and selenium can effectively prolong or enhance age-related declines in muscle function and strength (Veronese et al., 2014; Ter Borg et al., 2016; Fodor et al., 2020) (Table 1). Vitamins and fatty acids play a critical role in the prevention of sarcopenia through the inhibition of oxidative stress and relevant genes (Wang et al., 2021) (Table 1). In addition, a large number of phytochemicals such as polyphenols, polysaccharides, flavonoids, alkaloids, and triterpenoids may prevent and improve age-related sarcopenia through antioxidant and or anti-inflammatory (Li et al., 2004) (Table 1). Also, probiotics and intestinal flora have recently been considered to play a crucial role in the prevention of sarcopenia (An et al., 2019; Lahiri et al., 2019; Ni et al., 2019) (Table 1). For more detailed description, please refer to the excellent comments of Wang et al. and Nikawa et al. (Nikawa et al., 2021; Wang et al., 2021). Here, we only discuss some potential antioxidant substances or anti-inflammatory substances of interest for laboratory or preclinical studies to delay skeletal muscle aging and sarcopenia.

**TABLE 1:** The functions of potential therapeutic strategies for sarcopenia during aging.

Therapeutic strategies		Functions
Exercise		Improve muscle strength and increase muscle mass
Protein nutrients	Leucine (whey proteins)	Increase muscle metabolism (Dardevet et al., 2012)
	Carnosine (β-alanine)	ROS scavenging, antioxidant, anti-inflammatory, resist saccharification, neuroprotective properties (Bellia et al., 2011; Jukic et al., 2021)
Mineral matter **(Calcium, magnesium, selenium)**		Improve muscle performance and delay the decline of muscle strength (Veronese et al., 2014; Ter Borg et al., 2016; Fodor et al., 2020)
Vitamins **(VC, VD, VE)**		Eliminate both water-soluble and fat-soluble free radicals simultaneously (Geng et al., 2021), maintain muscle mass and strength; prevent or treat muscle atrophy (Kondo et al., 1992; Makanae et al., 2013; Nakamura et al., 2020)
ω-3 PUFAs **(ALA, EPA, DPA, DHA)**		Anti-inflammation, anti-aging, improving mitochondrial function, promoting myoblast differentiation and myotube fusion, enhancing muscle quality and strength (Smith et al., 2015; Chen et al., 2016; Custodero et al., 2018; Lovsletten et al., 2018; Tan et al., 2018; Xu et al., 2018)
Natural chemicals or plants	Polyphenols (Resveratrol, anthocyanins, phenolic Acids, Flavones)	Anti-inflammatory, antioxidant, improve mitochondrial quality, resist muscle atrophy (Salucci and Falcieri, 2020; Nikawa et al., 2021)
	*Ganoderma lucidum*	Inhibiting the production of ROS and lipid peroxidation, free radical scavenging, antioxidant, anti-aging, reduce muscle mass loss (Thyagarajan et al., 2006; Sanodiya et al., 2009; Zhonghui et al., 2014; Jatwani and Tulsawani, 2021)
Probiotics and intestinal flora		Enhance muscle mass and strength, anti-inflammatory (Ticinesi et al., 2019; Liu et al., 2021; van Krimpen et al., 2021)
Synthetic drugs	Anti-inflammatory Drugs (Thalidomide, tocilizumab, infliximab)	Preventing muscle atrophy via inhibition of inflammatory cytokines and their downstream effectors (Guasconi and Puri, 2008)
	Enobosarm	Induced muscle protein synthesis (Nikawa et al., 2021)
Stem cell therapy **(Mesenchymal stem cell)**		Increase skeletal muscle weight and muscle fiber cross-sectional area, activate resident skeletal muscle stem cells (Li et al., 2016; Wang et al., 2018)
Gene therapy **(miR-1, miR-206, miR-133a, miR-133b, miR-208b)**		Regulate inflammation and redox signals, regulate mitochondrial dysfunction and skeletal muscle regeneration (Barbiera et al., 2020)

Note: Whey protein, one of the primary proteins found in dairy products; VC, vitamin C; VD, vitamin D; VE, vitamin E; ALA, α-linolenic acid; EPA, eicosapentaenoic acid; DPA, docosapentaenoic acid; DHA, docosahexaenoic acid; ω-3, Omega-3; PUFAs, Polyunsaturated fatty acids; ROS, reactive oxygen species.

### Carnosine

Carnosine is a dipeptide that is synthesized *in vivo* from β-alanine and l-histidine in skeletal muscle (Jukic et al., 2021). Although its physiological role is not fully clear, carnosine is a nonenzymatic free radical scavenger and native antioxidant with neuroprotective and anti-inflammatory properties (Bellia et al., 2011). For example, carnosine can scavenge ROS and α-β-unsaturated aldehydes produced by lipid peroxidation as a result of oxidative stress (Jukic et al., 2021). Carnosine alleviates diabetic nephropathy and delay aging as it protects mesangial cells and podocytes (Jukic et al., 2021). Carnosine also resists saccharification by chelating divalent metal ions (Jukic et al., 2021). The amino acid β-alanine, as its main component, is often used as a nutritional supplement for athletes to improve exercise and muscle contraction (Jukic et al., 2021). In addition, its many beneficial effects are well confirmed, including pH buffering capacity, heavy metal chelating and anti-saccharification activity (Hipkiss, 2009; Vistoli et al., 2012; Ghodsi and Kheirouri, 2018). It has been shown that long-term oral β-alanine can significantly increase the content of carnosine in skeletal muscle, which is the main site of carnosine generation and accumulation in the body (Derave et al., 2010). Similarly, another study indicated that β-alanine intake can effectively enhance muscle carnosine content, thereby improving exercise capacity in older subjects (del Favero et al., 2012). A large number of studies have provided evidence for the beneficial effects of carnosine supplements on cytokine release, and the excessive expression of the pro-inflammatory IL-6, IL-8 and TNF-α is involved in the inflammatory process in digestive system, especially in intestine, which can induce inflammatory bowel disease (Katsanos and Papadakis, 2017). A few studies have demonstrated that carnosine inhibits the secretion and expression of IL-8 stimulated by hydrogen peroxide and TNF-α (Son et al., 2008). Altogether, these encouraging results demonstrate that supplementation with carnosine or its precursor substances may improve muscle strength, endurance, and function in the elderly. Therefore, considering the beneficial characteristics and its clinical application, carnosine is considered as a highly effective therapy without side effects.

### Ganoderma lucidum

*Ganoderma lucidum* (*G. lucidum*, *Lingzhi*) is a large white-rot macrofungus of basidiomycete, which is widely used as “the mushroom of immortality” in China. In Chinese folklore, it is considered to be a panacea for various diseases. The spores, basidiocarp, and mycelia of *Ganoderma lucidum* are reported to contain about 400 different biologically active compounds, mainly including triterpenoids, polysaccharides, sterols, steroids, nucleotides, fatty acids, peptides, proteins and trace elements, which makes *Ganoderma lucidum* have various pharmacological effects (Sanodiya et al., 2009). *Ganoderma lucidum* has been applied for thousands of years as an elixir, but its anti-aging effects still unrevealed, especially in skeletal muscle. Aging is associated with oxidation stress, free radical product and immunoregulation. It is currently believed that *Ganoderma lucidum* can extend lifespan through inhibiting the production of ROS, lipid peroxidation and advanced oxidation protein products (Sanodiya et al., 2009). In addition, it has antioxidant activity and immunomodulatory via enhancing free radical scavenging activity and diminishing iron antioxidant capacity (Sanodiya et al., 2009). The biologically active components of *Ganoderma lucidum* that have anti-aging related functions include triterpenoids, polysaccharides, and peptides, which all have antioxidant properties. For example, *Ganoderma lucidum* triterpenes can elevate the levels of SOD, GPx, CAT, and GSH in brain and liver tissues, protect the body from protein and lipid peroxidation that induced by oxidative stress (You and Lin, 2003). In addition, *Ganoderma lucidum* polysaccharides (GLPS-I, GLPS-II, GLPS-III, GLPS-IV) can increase hydroxyl and 2,2-diphenyl-1-picrylhydrazil radical scavenging activities and metal chelating activities (Shi et al., 2013; Wang et al., 2017). A study continuously administered GLPS (50, 100 and 200 mg/kg/day) to male mice for 28 days and found that GLPS could enhance the antioxidant enzymes activity in mouse skeletal muscle, including SOD, GPx, CAT, and reduce the level of malondialdehyde, indicating that the supplementation of GLPS could reduce exercise-induced oxidative stress in skeletal muscle (Zhonghui et al., 2014). *Ganoderma lucidum* peptide diminish lipid peroxidation primarily by scavenging hydroxyl radicals (^•^OH), which are the main activated ROS that participate in lipid oxygenation (Thyagarajan et al., 2006). *Ganoderma lucidum* peptide prevents lipid metabolism-related domino reaction through response with free ^•^OH (Sun et al., 2004). In addition, *Ganoderma lucidum* peptide can eliminate excessive free radicals and chelate metals ions to minimize metal-induced oxidation (Sun et al., 2004). *Ganoderma lucidum* polysaccharide peptide, the combination of polysaccharide and peptide, can reduce the oxidase NADPH and the NADPH-dependent ROS production and diminish malonaldehyde levels in a kidney ischemia-reperfusion model, while enhancing the activities of antioxidant enzymes SOD, Mn-SOD, GSH, GSH-px and CAT (Sun et al., 2004; Wang et al., 2017). A recent study showed that supplementation with an aqueous extract of *Ganoderma lucidum* attenuated the loss of muscle mass in rats through preventing oxidative stress and regulating myogenesis markers under low-pressure hypoxia (Jatwani and Tulsawani, 2021). Nevertheless, the anti-aging effect of *Ganoderma lucidum* is still a mystery, and the potential anti-aging mechanism of its clinical application remains to be revealed.

### Omega-3 polyunsaturated fatty acid

Polyunsaturated fatty acids (PUFAs) are a class of fatty acids whose molecular structure is featured by two or more double bonds. PUFAs family mainly includes Omega-3 (ω-3) and Omega-6 (ω-3) family. ω-3 PUFAs are distinguished by the fact that the first double bond is located on the 3rd carbon. ω-3 PUFAs are mainly α-linolenic acid (ALA), eicosapentaenoic acid (EPA), docosapentaenoic acid (DPA) and docosahexaenoic acid (DHA). Among them, EPA, DPA and DHA are regarded as long-chain PUFAs, while ALA is recognized as a short-chain PUFAs (Vannice and Rasmussen, 2014). Accumulating evidence showed that ω-3 PUFAs have the functions of anti-inflammatory, anti-aging, anti-cancer, and have potential effects on regulating blood lipids and blood pressure, improving cardiovascular and cerebrovascular diseases, improving insulin resistance and diabetes treatment.

A recent meta-analysis confirmed a reduced c-reactive protein and IL-6 with ω-3 PUFAs supplementation in older adults (Custodero et al., 2018). Continuous supplementation of EPA and DHA in the elderly for 4 weeks can significantly reduce the level of IL-1β, IL-6 and TNF-α, and this effect is more obvious after 8 weeks (Tan et al., 2018). As mentioned previously, chronic low-grade inflammation is considered to play a key role in the progression of sarcopenia. ω-3 PUFAs are potential therapeutics for the treatment of sarcopenia owing to their anti-inflammatory and anti-aging characteristics. Hence, the suppressing of this low-grade inflammation is generally recognized as a potential mechanism by which ω-3 PUFAs may antagonize sarcopenia. For example, ω-3 PUFAs supplementation for 6-months in healthy older adults dramatically enhanced muscle volume and strength compared with the placebo group (Smith et al., 2015). Additional evidence suggests that ω-3 PUFAs also might exert anabolism effects on muscle by activating mTOR signaling and reducing insulin resistance (Dupont et al., 2019). As mentioned above, the activation and functions of SMSCs are influenced by oxidative stress and inflammation during myogenesis, ω-3 PUFAs play a critical role in this process. For example, SMSCs were incubated with 50 mM DHA for 2 days found that DHA treatment resulted in longer multinucleated myotubes and higher myotube fusion rate compared to control (Xu et al., 2018). Treatment in human SCMSs with 100 μM EPA for 24 h increased cellular oxygen consumption rate, basal respiration, maximal respiration and proton leak, suggesting that EPA improves mitochondrial function during myogenesis (Lovsletten et al., 2018). Additionally, co-culture of C2C12 myotubes with palmitic acid, EPA and DHA for 16 h can significantly inhibit the palmitic acid-induced expression of proinflammatory cytokine (Chen et al., 2016). In conclusion, ω-3 PUFAs are promising in the prevention or treatment of sarcopenia, either alone or in combination with classical treatment strategies. In addition, combined with exercise intervention, the supplementation of ω-3 PUFAs may enhance the gains in muscle mass and function obtained through exercise intervention.

### Vitamins

Vitamins have been shown to play a critical role in preventing muscle atrophy by inhibiting oxidative stress and related genes. Vitamin C has the potential to treat or prevent muscle atrophy in mice, and its deficiency leads to muscle atrophy, while the muscle atrophy recovered after about 3 months of continuous vitamin C supplementation, which was attributed to the excessive production of ROS and the elevated expression of muscle atrophy marker genes MAFbx and MuRF1 (Takisawa et al., 2019). In addition, oral vitamin C diminished overload-induced skeletal muscle hypertrophy in male Wistar rats (Makanae et al., 2013). Dietary vitamin C supplementation helps reduce age-related muscle loss in the elderly (Lewis et al., 2020). The lack of Vitamin D might lead to muscle atrophy, while oral vitamin D prevents immobilization-induced muscle atrophy in mice through the vitamin D receptor (Nakamura et al., 2020). In addition, as an active form of Vitamin D, 1,25-dihydroxyvitamin D deficiency can lead to the development of age-related sarcopenia by inducing oxidative stress, muscle cell senescence and senescence-associated secretory phenotype, and inhibiting skeletal muscle regeneration (Yu et al., 2021). The possible mechanisms of vitamin D are associated with the elevated MAFbx expression and protein degradation (Wang et al., 2021). Vitamin E also prevented hindlimb muscle atrophy induced by immobilization in Wistar rats (Kondo et al., 1992). As one of the subgroups of vitamin E, tocopherols have beneficial effects in various pathophysiological models (i.e., hindlimb suspension, exercise, and ischemic perfusion injury) by reducing oxidative stress, inflammation, and atrophy while increasing regenerative capacity (Chung et al., 2018). Supplementation with α-tocopherol before and during muscle atrophy reduces hindlimb-induced slow twitch muscle atrophy, contributing to an increase in type I and type IIA muscle fiber size and a decrease in muscle proteolysis rate (Servais et al., 2007). Meanwhile, a study found that supplementation with α-tocopherol at a dose of 800 IU for 48 days decreased the expression of oxidative stress markers in both young and older sedentary men (Margaritis et al., 1997). A study also showed that α-tocopherol supplementation 1 h prior to exercise under hypoxic conditions reduced cellular damage and inflammation in healthy young men (Santos et al., 2016). Notably, combinations of different vitamins may exert more beneficial effects. For example, parallelly use of vitamin C and E is effective in eliminating both water- and fat-soluble free radicals simultaneously, and protecting cell membranes from oxidative damage induced by free radicals (Geng et al., 2021). Oral vitamin D and E supplementation can maintain muscle mass and muscle strength, and further improve the life quality of patients with sarcopenia (Bo et al., 2019). Taken together, vitamins have therapeutic potential to induce muscle hypertrophy and inhibit muscle atrophy by mechanisms that inhibit ROS-mediated oxidative stress, activate mitochondrial biogenesis, and suppress the expression of genes associated with muscle atrophy.

## Concluding remarks

Sarcopenia is one of the most severe geriatric syndromes. Under the trend of global aging, there is an urgent need to develop effective preventive and therapeutic measures. Although current research have clarified numerous pathophysiological factors in sarcopenia, such as the currently widely accepted mechanisms of oxidative stress and inflammation, and several therapeutic strategies have been proved to be effective in the treatment of sarcopenia. However, the majority of studies have been conducted in *in vitro* and *in vivo* models, while studies on humans are lacking. Overall, the molecular mechanisms that affect sarcopenia remain elusive. Focusing on the currently pathogenic mechanism of sarcopenia, it may be a long-term work to find, research and extensively apply antioxidants and anti-inflammatory substances, such as the synthesis of antioxidant and/or anti-inflammatory drugs. Currently, exercise is the only treatment that has been proven effective for delaying muscle loss. A combination of exercise, potential antioxidant therapy, and anti-inflammatory therapy probably is the most efficient strategy to prevent or treat sarcopenia during aging.
